# Reference Database Selection Influences Both Taxonomic Annotation and Ecological Inference in Arbuscular Mycorrhizal Fungal Communities

**DOI:** 10.3390/jof12090703

**Published:** 2026-09-20

**Authors:** Yajie Liu, Na Li, Xiaoshuang Ma, Kaiyuan Zhang, Nianci Li, Chunxue Yang

**Affiliations:** College of Landscape Architecture, Northeast Forestry University, Harbin 150040, China; 16653316925@163.com (Y.L.); 13835356415@163.com (N.L.); axiye1966@126.com (X.M.); zky199911@163.com (K.Z.); linianci66yeah@163.com (N.L.)

**Keywords:** arbuscular mycorrhizal fungi, high-throughput sequencing, database comparison, community profiling, soil factor fitting

## Abstract

Arbuscular mycorrhizal (AM) fungi are keystone microorganisms in soil ecosystems, and their community composition plays a crucial role in regulating ecosystem functions. Although high-throughput sequencing is widely used to characterize their communities, the selection of reference databases for sequence annotation remains inconsistent. To assess database performance at the species level, rhizosphere soil and root samples from four plant species with contrasting communities were subjected to amplicon sequencing. Taxonomic annotations generated using the MaarjAM, SILVA, and NCBI databases were compared. NCBI provided the highest taxonomic resolution, identifying 18 genera and 108 species, including *Rhizophagus intraradices* and *Funneliformis mosseae*, with assignments supported by spore isolation and nomenclatural verification. In contrast, MaarjAM recovered the most ASVs but contained numerous virtual taxa, whereas SILVA showed limited annotation depth and fewest assignments. Comparative analyses showed that NCBI was more sensitive in distinguishing samples based on alpha diversity and community structure and produced species distributions more consistent with ecological expectations. Although core metabolic profiles were conserved across databases, suggesting functional redundancy that may contribute to ecosystem stability, database selection influenced relationship interpretation between environmental variables and communities. Overall, NCBI is recommended for species-level annotation and community analysis, whereas inappropriate database selection may bias biodiversity assessment and ecological inference.

## 1. Introduction

Arbuscular mycorrhizal (AM) fungi, a functionally pivotal microbial group widely distributed in soil ecosystems, are capable of forming symbiotic associations with the majority of vascular plants [1]. Through these symbiotic interactions, AM fungi enhance host plant acquisition of mineral nutrients, particularly phosphorus, as well as water uptake [2,3,4], while improving plant tolerance to both biotic and abiotic stresses [5,6]. Moreover, their extraradical hyphae and associated exudates contribute to soil physicochemical improvement and regulate the availability of essential elements [7,8]. Glomalin-related soil proteins, in particular, play an important role in promoting soil aggregation and maintaining soil quality [9]. Collectively, these functions highlight the indispensable role of AM fungi in supporting ecosystem stability.

In natural ecosystems, AM fungi rarely exist as isolated species. Instead, they colonize plant roots and persist in rhizosphere soils as complex communities shaped by local environmental conditions [10,11]. Interactions among community members, including competition and cooperation for resources, play important roles in determining community functioning [12]. Therefore, variations in species composition and diversity may directly influence the ecological functions of AM fungal communities. Accurate characterization of community structure and dynamics is consequently essential for understanding their ecological roles. Traditional approaches, such as morphological identification based on spore characteristics, have provided valuable insights into AM fungal diversity. However, these methods are constrained by difficulties in cultivation, limited morphological traits, and insufficient sampling depth [13], making them inadequate for accurately reflecting community composition in environmental samples. Although terminal restriction fragment length polymorphism (T-RFLP) partially addressed some of these limitations, the co-migration of fragments with identical lengths can substantially underestimate species diversity [14]. Recent advances in high-throughput sequencing have enabled amplification and large-scale sequencing of AM fungal marker genes directly from environmental samples, providing sensitive and high-resolution characterization of AM fungal community structure and facilitating detailed investigations of AM fungal ecology and species diversity [15,16].

Currently, although high-throughput sequencing technologies are widely used to investigate AM fungal communities, analytical outcomes are strongly influenced by methodological choices, particularly the selection of molecular markers and reference databases. Several genetic markers, including SSU, ITS, and LSU regions, have been employed in AM fungal community studies [17,18,19]. Among these markers, the SSU rRNA region has become one of the most commonly used targets because it effectively discriminates major AM fungal lineages while providing a favorable balance of sequence conservation, taxonomic resolution, database availability, and technical feasibility [20,21]. Reference database selection represents another critical factor affecting community analyses; however, systematic comparisons of database performance remain scarce. This knowledge gap not only influences sequence annotation but also affects subsequent ecological interpretations and the resolution of ecological questions. Among the currently available databases, MaarjAM is the most frequently used, whereas SILVA is often applied independently or for preliminary screening of target sequences, and some studies also utilize the NCBI database [20,22,23]. MaarjAM is specifically developed for AM fungi and provides carefully curated reference sequences, although its taxonomic coverage remains relatively limited [24]. In contrast, SILVA contains SSU rRNA sequences covering a broad range of eukaryotic taxa, offering comprehensive coverage but sometimes insufficient taxonomic resolution for AM fungi [25]. As a comprehensive sequence repository, NCBI contains the largest volume of available sequence data; however, the heterogeneous quality of deposited sequences may introduce uncertainty into taxonomic assignments [26]. Given differences in sequence sources and annotation criteria among the three databases, database selection may substantially influence analytical outcomes. Therefore, evaluating their performance and limitations is essential, particularly in terms of database characteristics, species-level annotation reliability, and downstream effects on community analyses and ecological interpretation.

To evaluate the effects of database selection on species-level taxonomic annotation and ecological inference, this study selected four common mycorrhizal plant species representing distinct ecological niches, aiming to obtain samples with contrasting AM fungal community compositions across ecological gradients. Rhizosphere soil and root samples were collected and subjected to SSU rRNA amplicon sequencing, and the resulting sequences were taxonomically annotated against the MaarjAM, SILVA, and NCBI reference databases. Subsequently, analyses were conducted to evaluate the influence of database selection on AM fungal community characterization and on the interpretation of relationships between environmental variables and community structure. In addition, spore isolation and taxonomic verification were employed to assess the reliability of taxonomic assignments generated by each database. We hypothesized that

(1) databases with broader taxonomic coverage, particularly NCBI, would provide higher species-level taxonomic resolution and greater sensitivity in characterizing AM fungal community composition; and
(2) predicted functional profiles and inferred relationships between environmental factors and AM fungal community composition would vary among databases due to differences in taxonomic assignments.

## 2. Materials and Methods

### 2.1. Study Site and Sample Collection

The study was conducted in the Songnen Grassland of eastern Eurasia (46°04′–46°46′ N, 125°37′–125°88′ E). Four AM fungal host species representing distinct ecological niches were selected: *Lythrum salicaria* (wetland and low-lying saline habitats), *Medicago sativa* (arid, semi-arid, and mildly saline grasslands), *Inula japonica* (moist meadows and lakeside wetlands), and *Taraxacum mongolicum* (widely occurring across the study area).

Within the study area, three plots were randomly established for each of the four plant species, with a minimum distance of 50 m between plots to provide three independent biological replicates per species. At each of the 12 plots, five healthy individuals were selected using a five-point sampling design. After removing surface litter, soil cores (0–30 cm depth) containing sufficient fine roots were collected, sealed, and transported to the laboratory at 4 °C. Subsequently, roots were separated from the soil, and rhizosphere soil within 2 mm of the root surface was collected. Roots and soil samples from the same plot were pooled separately, resulting in 12 root samples and 12 soil samples. The soil samples were then divided into two portions for high-throughput sequencing and soil physicochemical analyses, while root surfaces were gently cleaned to remove adhering soil particles. Finally, root samples and soil samples designated for sequencing were stored at −80 °C until DNA extraction.

### 2.2. High-Throughput Sequencing for Collected Samples

**Figure A1:**
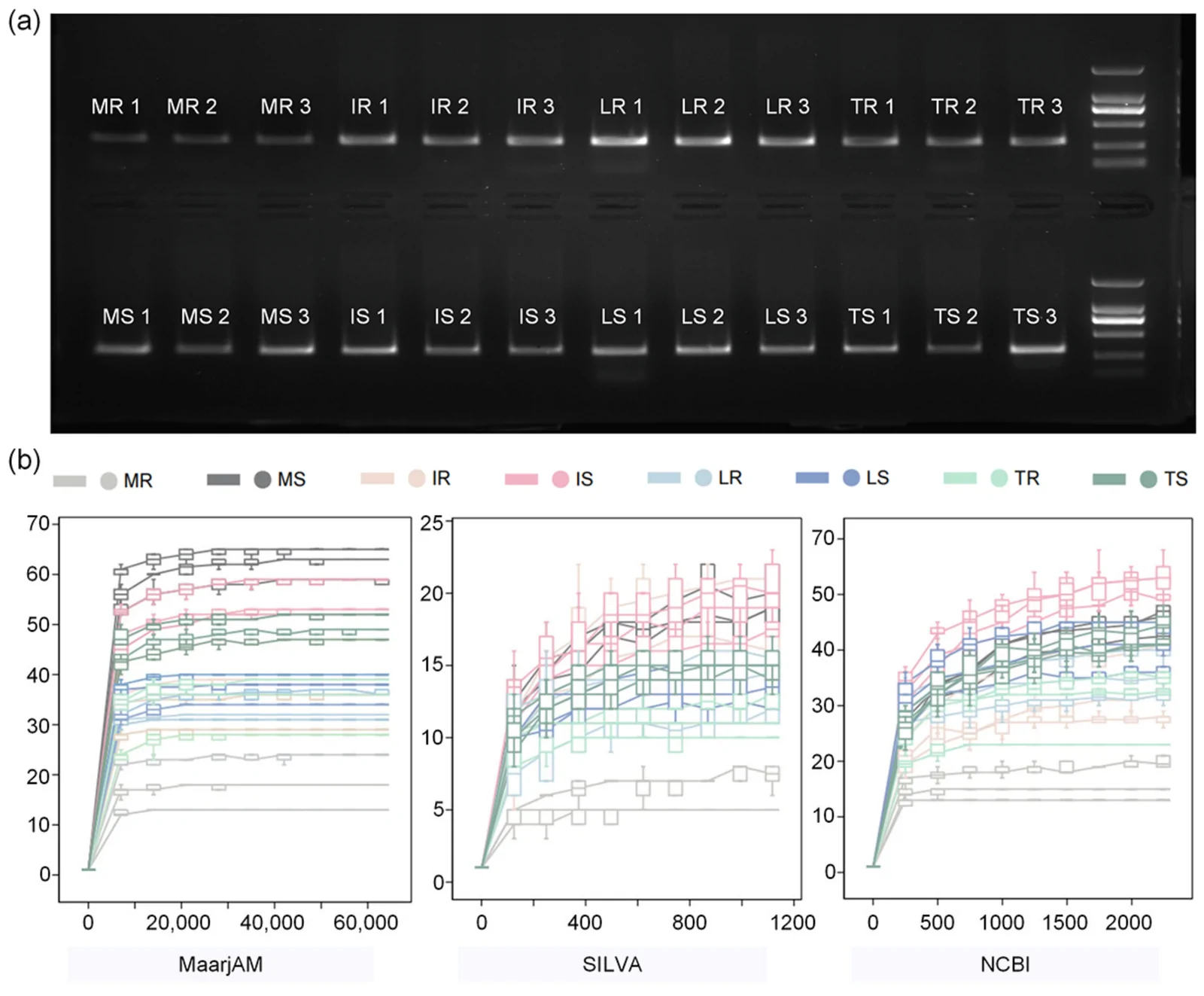
PCR amplification of AM fungal fragments and rarefaction analysis of sequencing data: (**a**) Agarose gel electrophoresis of AM fungal fragments amplified from root and soil samples. (**b**) Rarefaction curves of AM fungal species after sequence processing and rarefaction. MR, *M. sativa* root; MS: *M. sativa* soil; IR: *I. japonica* root; IS: *I. japonica* soil; LR: *L. salicaria* root; LS: *L. salicaria* soil; TR: *T. mongolicum* root; TS: *T. mongolicum* soil.

DNA was extracted from 0.2 g of each rhizosphere soil and root sample (24 samples in total) using the Omega Mag-Bind Soil DNA Kit (M5635-02, Omega Bio-tek, Inc., Norcross, GA, USA). The primer pair AMV4.5NF (5′-AAGCTCGTAGTTGAATTTCG-3′) and AMDGR (5′-CCCAACTATCCCTATTAAATCAC-3′) was then used to amplify an approximately 280 bp fragment of the AM fungal SSU rRNA region (Figure A1a). The 25 μL PCR reaction mixture contained 5 μL of 5× reaction buffer, 5 μL of 5× GC buffer, 2 μL of dNTPs (2.5 mM each), 1 μL of forward primer (10 μM), 1 μL of reverse primer (10 μM), 2 μL of DNA template, 8.75 μL of ddH_2_O, and 0.25 μL of Q5 DNA Polymerase. The amplification procedure consisted of an initial denaturation at 98 °C for 3 min, followed by 35 cycles of denaturation at 98 °C for 30 s, annealing at 50 °C for 30 s, and extension at 72 °C for 45 s, with a final extension at 72 °C for 5 min. The products were subsequently maintained at 12 °C until further processing. The amplified fragments were purified using the Axygen Gel Extraction Kit and quantified using the Quant-iT PicoGreen dsDNA Assay Kit with a Microplate Reader (M5635-02, Omega Bio-tek, Inc., Norcross, GA, USA). Thereafter, libraries were constructed using the TruSeq Nano DNA LT Library Prep Kit, and paired-end sequencing was performed on the Illumina NovaSeq platform (PE250) by Shanghai PersonalBio Co., Ltd. (Shanghai, China). In this study, each sample generated more than 80,000 raw sequences. Subsequently, the raw reads were processed using QIIME 2 (version 2024.5). Primers were removed using the Cutadapt plugin, followed by primer removal, quality control, denoising, read merging, chimera elimination, and generation of representative sequences using the DADA2 plugin (qiime dada2 denoise-paired) [27]. During sequence processing, the allowed barcode mismatch was set to 0. The maximum primer mismatch ratio was set to 0.2, with a minimum matching coverage of 90% of the primer length. Low-quality bases were trimmed using the parameter trunc-q = 2. Forward and reverse reads were truncated to uniform lengths using p-trunc-len-f = 221 and p-trunc-len-r = 229, respectively. Read merging was performed with a minimum overlap length of 8 bp and a maximum of 4 mismatches allowed within the overlap region. Sequences that failed to meet these criteria were removed, and chimeric sequences were subsequently eliminated using the consensus method.

### 2.3. Sequence Alignment Against Different Reference Databases and Dataset Preparation

Following the generation of merged amplicon sequence variant (ASV) tables, taxonomic annotation was performed using the classify-sklearn algorithm implemented in QIIME2 against three reference databases: MaarjAM 2019-10, SILVA release 138.1, and NCBI nt. The confidence threshold was set to the default value of 0.7, which has been widely applied in taxonomic annotation based on marker-gene amplicon sequencing [28]. Because the MaarjAM database exclusively contains sequences assigned to Glomeromycota, all annotated taxa were retained as AM fungal taxa for subsequent analyses. In contrast, annotations generated using the SILVA and NCBI database included numerous non-target taxa due to their broader taxonomic coverage; therefore, non-AM fungal lineages were removed before downstream analyses. Specifically, AM fungal sequences were identified as those assigned to Glomeromycetes (within Mucoromycota in Eukaryota), whereas all other taxonomic groups were excluded. Subsequently, to ensure an equal number of sequencing reads across samples and to follow standard analytical procedures, rarefaction was independently performed for all 24 samples within each database-specific dataset. The rarefaction depth was set at 95% of the minimum sequence count among samples. Rarefaction curves showed that observed species richness gradually approached a plateau with increasing sequencing depth, indicating that the rarefied datasets captured the majority of community diversity (Figure A1b). The resulting datasets were used for taxonomic composition analysis, functional pathway prediction, and downstream analyses at the species level.

### 2.4. Validation of Database Annotation Reliability

To evaluate the reliability of species-level annotations generated by different reference databases, both the nomenclatural validity and biological occurrence of annotated species were assessed. Nomenclatural validity was verified by querying species names against the MycoBank. To independently validate species occurrence, morphological identification of AM fungal spores was performed. Briefly, AM fungal spores (approximately 400–600 spores per sample) were extracted from 20 g of rhizosphere soil using the wet sieving and decanting method described by Gerdemann and Nicolson [29]. For each sample, 300 spores were randomly collected under a stereomicroscope (Leica MDG33, Leica Microsystems, Wetzlar, Germany) and mounted on slides containing lactoglycerol medium. Morphological characteristics, including spore diameter, wall structure, surface ornamentation, cytoplasmic contents, subtending hyphal features, and staining reactions in Melzer’s reagent, were subsequently examined using a microscope (Zeiss Axio Scope A1, Carl Zeiss Microscopy GmbH, Jena, Germany). Based on these traits, species identification was performed with reference to the taxonomic descriptions provided by Wang et al. [30], species descriptions available from the International Collection of (Vesicular) Arbuscular Mycorrhizal Fungi (INVAM), and relevant published literature.

### 2.5. Determination of Soil Physicochemical Properties and Enzyme Activities

To characterize environmental conditions across sampling sites, four categories of soil properties, including soil salinity, nutrient status, physical characteristics, and enzyme activities, were determined. Soil physical properties included soil water content, measured according to Bao [31], clay fraction, determined using the hydrometer method [32], and the proportion of water-stable aggregates (>0.25 mm), assessed by wet sieving [33]. Other soil parameters were analyzed following the methods described by Bao [31] and Guan [34]. Specifically, soil salinity-related parameters included soil pH, electrical conductivity (EC), carbonate (CO_3_^2−^), and bicarbonate (HCO_3_^−^), with pH and EC measured using a PHS-3C pH meter and a DDS-11A conductivity meter, respectively, and carbonate and bicarbonate concentrations quantified using indicator-neutralization titration. For soil nutrient analyses, soil organic matter (SOM) was determined using the potassium dichromate volumetric method, while total nitrogen (TN) and total phosphorus (TP) were measured using the semi-micro Kjeldahl method and NaOH fusion–molybdenum-antimony anti-colorimetry, respectively. Enzyme activities, including sucrase, urease, catalase, and alkaline phosphatase (ALP), were assayed by 3,5-dinitrosalicylic acid colorimetry, sodium phenate–sodium hypochlorite colorimetry, potassium permanganate volumetric method, and disodium phenyl phosphate colorimetry, respectively. All measurements were conducted using the 12 rhizosphere soil samples, with three biological replicates corresponding to the three sampling plots of each plant species.

### 2.6. Statistical Analysis

One-way ANOVA and repeated-measures ANOVA were performed using IBM SPSS Statistics 25.0. For one-way ANOVA, significant differences among groups were determined using Duncan’s multiple range test. Repeated-measures ANOVA was applied to assess within-subject effects among different database-derived datasets, followed by pairwise comparisons with Bonferroni correction. Subsequently, comprehensive statistical analyses of AM fungal communities were conducted using methods commonly applied in microbial ecology. Alpha-diversity indices were calculated using the “qiime diversity alpha” command in QIIME2. Non-metric multidimensional scaling (NMDS), random forest analysis, and niche breadth calculations were conducted in R using the vegan, mlr3, and spaa packages, respectively, with normalized data used for NMDS and log10-transformed data used for random forest analysis. In addition, the effects of reference database selection on functional prediction and environmental association analyses were evaluated. Functional pathway prediction was performed using PICRUSt2 (version 2.2.2-b) based on AM fungal sequences assigned by each of the three databases. The MetaCyc database was used for pathway prediction, and predicted pathway abundances were normalized to one million functional units (PFU) before statistical comparisons. Variance partitioning analysis (VPA) and Mantel tests were conducted using the Vegan package to evaluate the relationships between soil environmental factors and AM fungal community composition. For VPA, dimensionality reduction was first performed separately for the four categories of soil properties to balance the number of samples with the number of environmental variables. The first two axes from each category were then extracted and used together with the community data generated from each reference database. Moreover, to minimize the potential influence of differences in the number of database-derived AM fungal sequences, all downstream analyses were conducted separately for the three database-specific datasets. Data visualizations, including line charts, bar charts, boxplots, Venn diagrams, and stacked bar charts, were generated using the online platform ChiPlot https://www.chiplot.online/ (accessed on 15 February 2026). Final figure assembly and layout were completed using Adobe Photoshop CC 2019.

## 3. Results

### 3.1. Soil Conditions at the Growth Sites of the Four Plant Species

Significant differences in rhizosphere soil properties were observed among the four plant species (Table 1). For soil salinity-related parameters, pH, EC, CO_3_^2−^, and HCO_3_^−^ were highest in *I. japonica* (8.06, 0.19, 0.25, and 0.58, respectively), whereas no significant differences were observed between *M. sativa* and *L. salicaria* (*p* > 0.05). Regarding soil nutrient status, *M. sativa* exhibited the highest SOM (78.65 g·kg^−1^) and TN (2.03 g·kg^−1^), while SOM was lower in *L. salicaria* and TN was lowest in *T. mongolicum*. TP was significantly lower in *I. japonica* (*p* < 0.05). Soil physical properties also varied among samples. Soil moisture content was highest in *I. japonica* (9.22%) and lowest in *T. mongolicum* (6.32%). The proportion of water-stable aggregates was greatest in *M. sativa* (52.90%), significantly exceeding that of the other species (*p* < 0.05), while clay content was highest in *I. japonica*. In addition, soil enzyme activities differed markedly among plant species. *M. sativa* showed the highest activities of catalase (2.55 mL·g^−1^·20 min^−1^), urease (1.70 mg·g^−1^·d^−1^), and sucrase (0.15 mg·g^−1^·d^−1^). ALP activity was highest in *M. sativa* (2.58 mg·g^−1^·d^−1^) and *T. mongolicum* (2.74 mg·g^−1^·d^−1^), whereas *L. salicaria* consistently exhibited low enzyme activities. Overall, *M. sativa* rhizosphere soils were characterized by higher nutrient content and a greater proportion of water-stable aggregates, *I. japonica* was associated with relatively saline conditions, *L. salicaria* with nutrient-poor soils, and *T. mongolicum* with relatively high ALP activity. These differences provided environmental gradients for subsequent analyses.

### 3.2. Sequence Data Processing and Taxonomic Assignment

**Figure 1:**
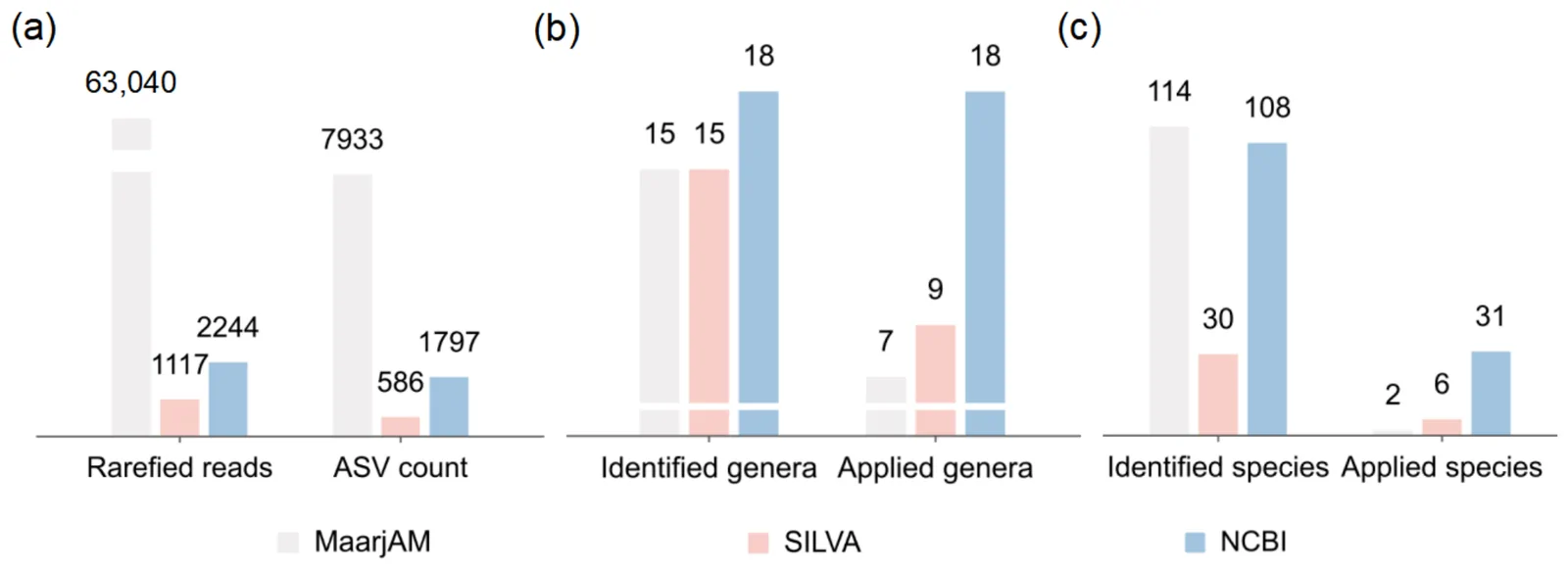
Basic characteristics of sequence annotation across different databases: (**a**) Sequence reads and ASV count after rarefaction. (**b**) Genera and applied genera identified by different databases. (**c**) Species and applied species identified under different databases.

As shown in Figure 1 and Table A1, taxonomic annotation results varied among databases, indicating that database selection influenced the number of taxa identified. Specifically, the numbers of AM fungal sequences and ASV count varied among datasets. The MaarjAM database recovered the highest numbers of AM fungal sequences (63,040) and ASVs (7933), followed by NCBI, whereas SILVA yielded the lowest values for both metrics (Figure 1a). At the genus level, NCBI annotated 18 genera, exceeding the numbers identified by the other two databases. Notably, all genera identified by NCBI represented formally recognized taxonomic units with practical applicability rather than virtual taxa, including nine genera uniquely detected by this database, such as *Entrophospora*, *Scutellospora*, and *Sclerocystis*. In contrast, although SILVA and MaarjAM each annotated 15 genera, many of their assignments lacked formal taxonomic identities and were represented by virtual taxa (Figure 1b). At the species level, NCBI identified 108 species, including 31 formally named species with practical applicability, such as *Rhizophagus intraradices* and *Funneliformis mosseae*, representing the highest number among the three databases. In comparison, although MaarjAM identified 114 species, a number comparable to that of NCBI, only two were formally recognized species with potential application value. This discrepancy may limit the interpretation of actual community composition despite its relatively high detection depth (Figure 1c).

**Figure A2:**
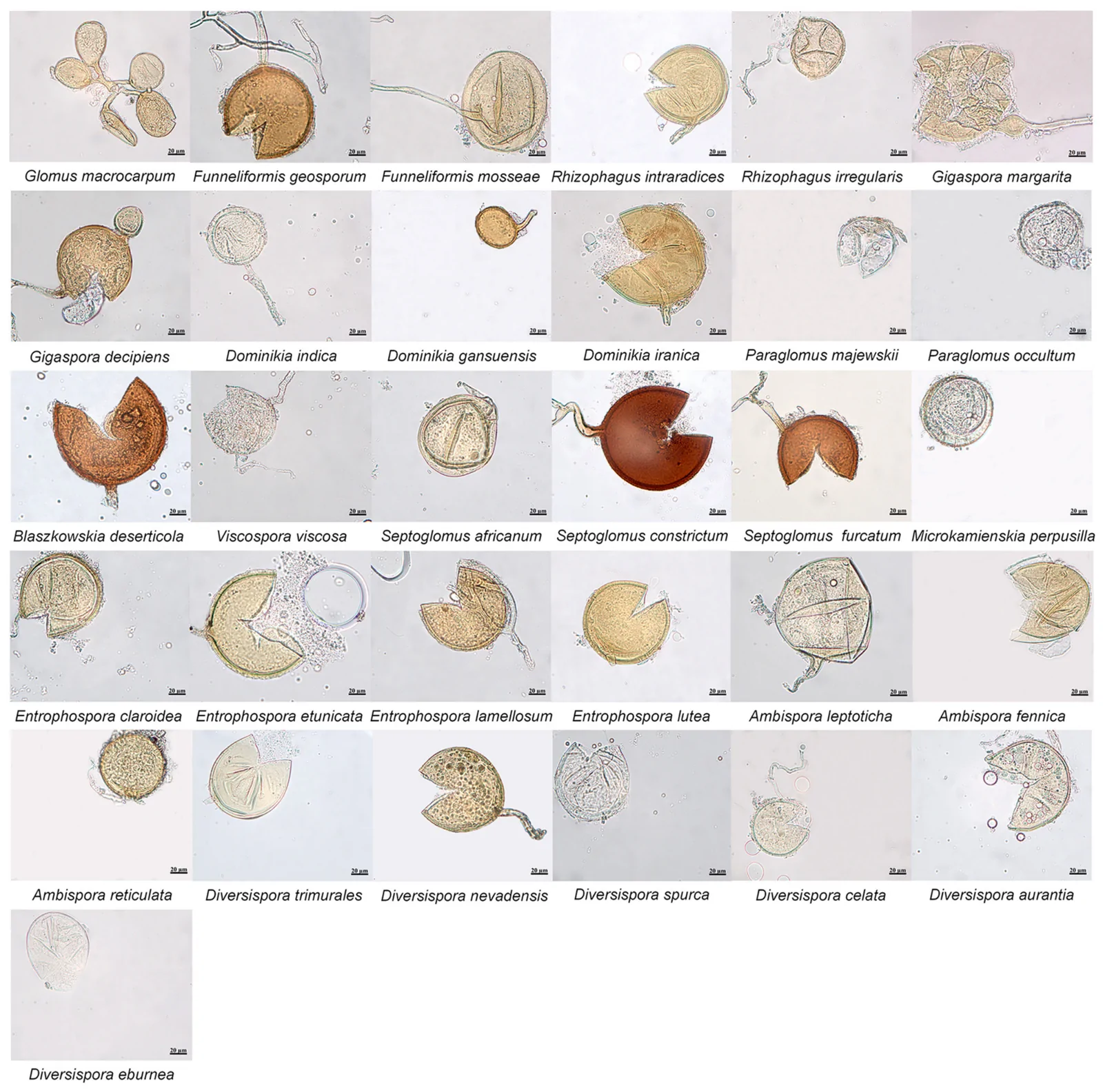
Spore isolation of AM fungal species identified by sequencing.

Reliability analyses of taxonomic annotations revealed that all three databases exhibited varying degrees of taxonomic inconsistencies with current AM fungal classification systems (Table A2). Specifically, 81.57% of the species-level assignments generated by MaarjAM were placed within *Glomus*, and both formally named species showed inconsistencies with current nomenclature. In the SILVA database, *Entrophospora* was assigned to *Claroideoglomus* based on an outdated taxonomic framework, and the proportion of inaccurate species names reached 33.33%. In contrast, 80.65% of the species names assigned by NCBI were consistent with current nomenclature, and the database contained numerous genera recognized in the latest classification system. The revised taxonomic assignments are presented in Table 2. Morphological identification of isolated spores further validated the database-derived annotations (Figure A2), with the diagnostic criteria for species identification summarized in Table A3. The species *Archaeospora schenckii*, which was exclusively annotated by SILVA, was not detected among the recovered spores. In contrast, most species identified by the other databases were supported by spore morphological observations, and their occurrence patterns were generally consistent with the sequencing data, with the exception of *Geosiphon pyriformis*. Furthermore, numerous morphologically distinct but taxonomically unresolved spore types were recovered, resulting in an estimated total species richness of approximately 110 taxa. This estimate was broadly consistent with the species richness inferred from the MaarjAM and NCBI databases. Overall, NCBI exhibited the highest proportion of taxonomically valid annotations and the greatest species-level resolution, whereas MaarjAM showed intermediate performance among the three databases.

### 3.3. Alpha Diversity of AM Fungal Communities Based on Different Database Annotations

**Figure 2:**
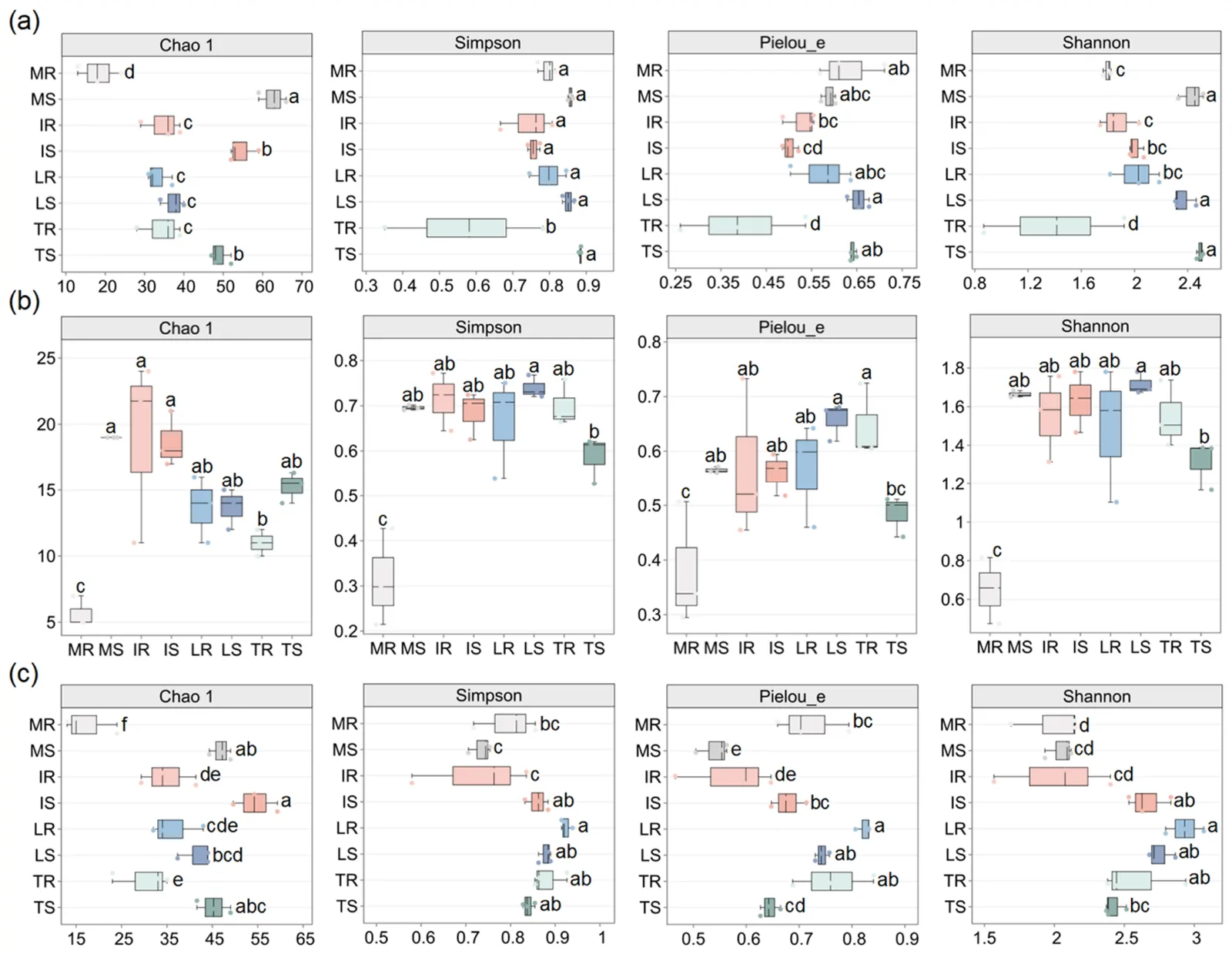
Alpha diversity based on different reference databases: (**a**) MaarjAM (**b**) SILVA (**c**) NCBI. Different letters within each subgraph indicate significant differences among groups (*p* < 0.05). MR, *M. sativa* root; MS: *M. sativa* soil; IR: *I. japonica* root; IS: *I. japonica* soil; LR: *L. salicaria root*; LS: *L. salicaria* soil; TR: *T. mongolicum* root; TS: *T. mongolicum soil*.

Spore-based morphological identification indicated that AM fungal community composition differed among samples. To compare the effects of reference database selection on community characterization, alpha diversity indices of AM fungal communities were calculated to assess differences in species richness, diversity, and evenness among database-derived datasets. One-way ANOVA revealed that MaarjAM-based annotations were able to distinguish differences in several indices (Figure 2a). SILVA-based annotations generally produced lower diversity estimates (Figure 2b), with reduced variation among samples, suggesting a limited capacity for detecting community differences. In comparison, NCBI-based annotations, supported by superior species-level detection capacity, revealed higher diversity levels and showed greater discrimination among samples across multiple diversity indices (Figure 2c). Subsequent repeated-measures ANOVA further quantified the effects of database selection on diversity estimates (Table A4). Although MaarjAM and NCBI did not differ significantly in Chao1 (Cohen’s d = −0.18) or Simpson (Cohen’s d = −0.58) indices (*p* > 0.05), indicating comparable performance for these diversity metrics, pairwise comparisons showed that SILVA differed significantly from NCBI across all four diversity indices (*p* < 0.05) and differed from MaarjAM in three indices (*p* < 0.05). Collectively, database-dependent species annotations could influence alpha diversity assessments. While NCBI exhibited stronger discriminatory power for sample characterization, MaarjAM also showed potential utility, whereas SILVA displayed relatively limited capacity for resolving community differences.

### 3.4. AM Fungal Community Structure Across Databases

**Figure 3:**
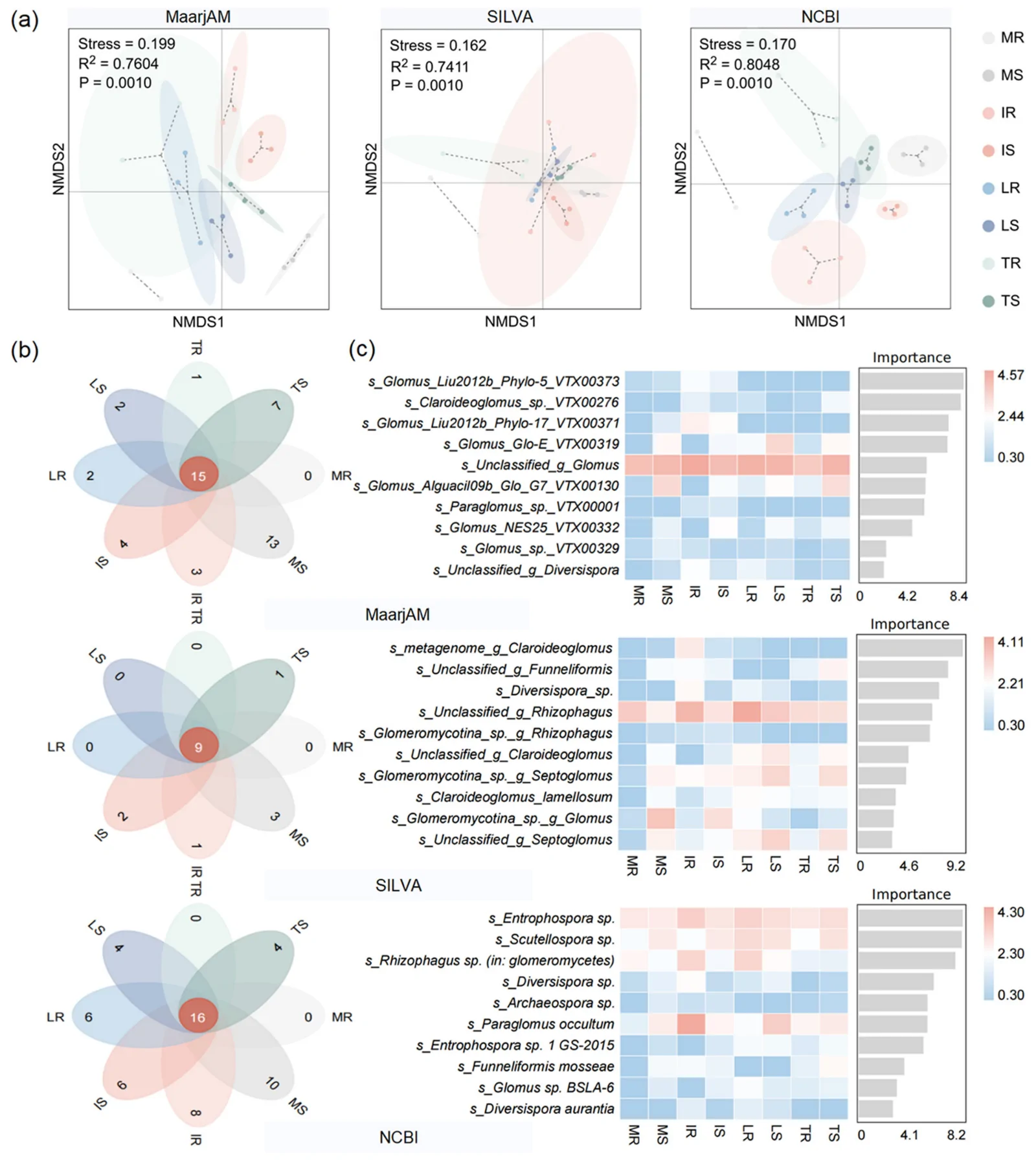
Structural differences in AM fungal communities based on different reference databases: (**a**) NMDS ordination of AM fungal community. (**b**) Venn diagram showing the overlap of AM fungal species, with numbers indicating core (shared among all samples) and unique species. (**c**) Identification of key AM fungal taxa using random forest modeling, with color gradients indicating differences in species abundance. MR, *M. sativa* root; MS: *M. sativa* soil; IR: *I. japonica* root; IS: *I. japonica* soil; LR: *L. salicaria root*; LS: *L. salicaria* soil; TR: *T. mongolicum* root; TS: *T. mongolicum soil*.

To elucidate the influence of reference database selection on AM fungal community structure profiling, NMDS ordination, Venn analysis, and random forest-based identification of key taxa were conducted. NMDS analysis revealed that all three database-derived datasets exhibited acceptable ordination reliability (stress < 0.2; *R*^2^ > 0.6; *p* = 0.001; Figure 3a), although their abilities to resolve differences in community composition among samples varied. Samples showed substantial overlap under MaarjAM and SILVA based annotations, whereas NCBI-based annotations provided clearer separation among different sample types, indicating a higher capacity for resolving community structure differences. Venn analysis revealed 28 unique AM fungal species under MaarjAM annotation (Figure 3b), with the number increasing to 38 under NCBI annotation, suggesting that different databases varied in their ability to identify rare and sample-specific taxa. Random forest analysis (Figure 3c) further demonstrated that the top-ranked species identified by MaarjAM were predominantly assigned to *Glomus*, and all represented virtual taxa, limiting their ecological interpretability. This limitation was partially alleviated in the other two databases. Notably, NCBI annotation identified three ecologically relevant key species, including *Paraglomus occultum*, *Funneliformis mosseae*, and *Diversispora aurantia*. Overall, supported by its superior taxonomic annotation performance, NCBI exhibited greater capacity to resolve AM fungal community structure and identify ecologically meaningful taxa.

### 3.5. Ecological Niche Differentiation and Functional Pathways Across Database Annotations

**Figure 4:**
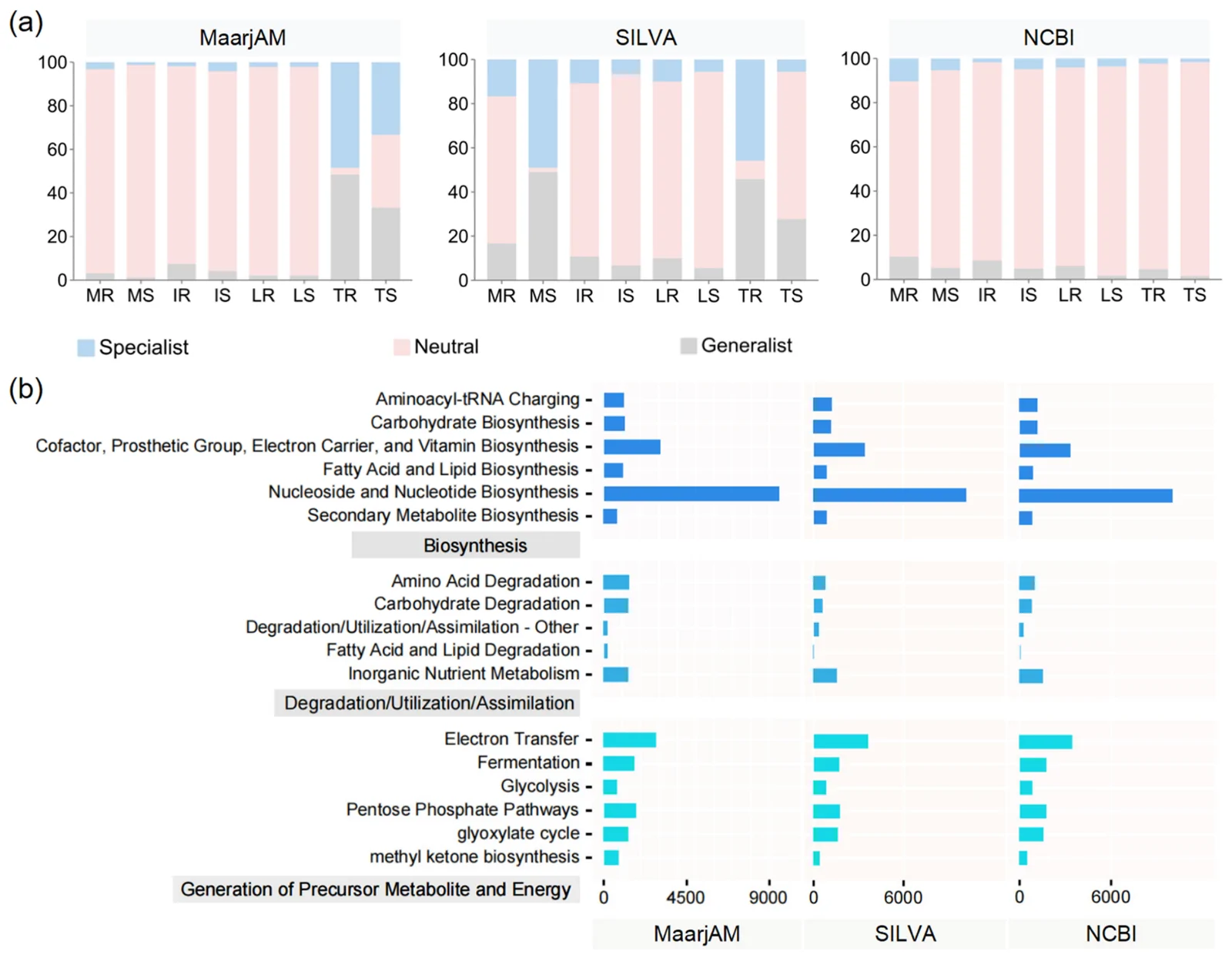
Species ecological specialization and functional profiles based on different reference databases: (**a**) Proportions of species with different ecological niche breadths. (**b**) Relative abundance of functional pathways predicted from AM fungal sequences annotated using three databases. MR, *M. sativa* root; MS: *M. sativa* soil; IR: *I. japonica* root; IS: *I. japonica* soil; LR: *L. salicaria root*; LS: *L. salicaria* soil; TR: *T. mongolicum* root; TS: *T. mongolicum soil*.

In natural communities, neutral species generally constitute the dominant component, whereas specialist and generalist taxa occur at lower proportions. In this study, taxa were classified based on niche breadth values as specialists (<1), generalists (>3), and neutral specialists (1–3), and their relative distributions are presented in Figure 4a. Specifically, analyses based on the MaarjAM and SILVA databases showed broadly similar patterns; however, deviations from theoretical expectations were observed in some samples, characterized by reduced proportions of neutral taxa. This suggests that differences in taxonomic annotation among databases may influence niche breadth estimation. Under MaarjAM annotation, neutral specialists accounted for only 3.16% and 33.33% in the roots and rhizosphere soils of *T. mongolicum*, respectively. In contrast, NCBI-based annotation consistently exhibited distribution patterns more closely aligned with ecological expectations across all samples and maintained clearer quantitative differences among sample types. These results indicate that the higher taxonomic resolution of NCBI may facilitate more accurate characterization of the ecological distribution patterns of AM fungal communities.

In addition, considering the ecological relevance of AM fungal functional potential, functional pathway profiles predicted from sequences annotated by the three databases were compared (Figure 4b). Although differences in pathway abundance were observed among databases, with MaarjAM showing higher overall values and the other two databases exhibiting comparable levels, the core metabolic framework remained highly conserved. The 17 most abundant pathways were consistently identified across all database-derived datasets, with similar relative proportions among pathways. Among these pathways, nucleotide and nucleoside biosynthesis in the biosynthesis category represented the dominant pathway across all datasets. Overall, these results indicate that the three databases showed comparable performance in characterizing the core metabolic potential of the studied AM fungal communities.

### 3.6. Relationships Between Soil Factors and AM Fungi Annotated from Different Database

**Figure 5:**
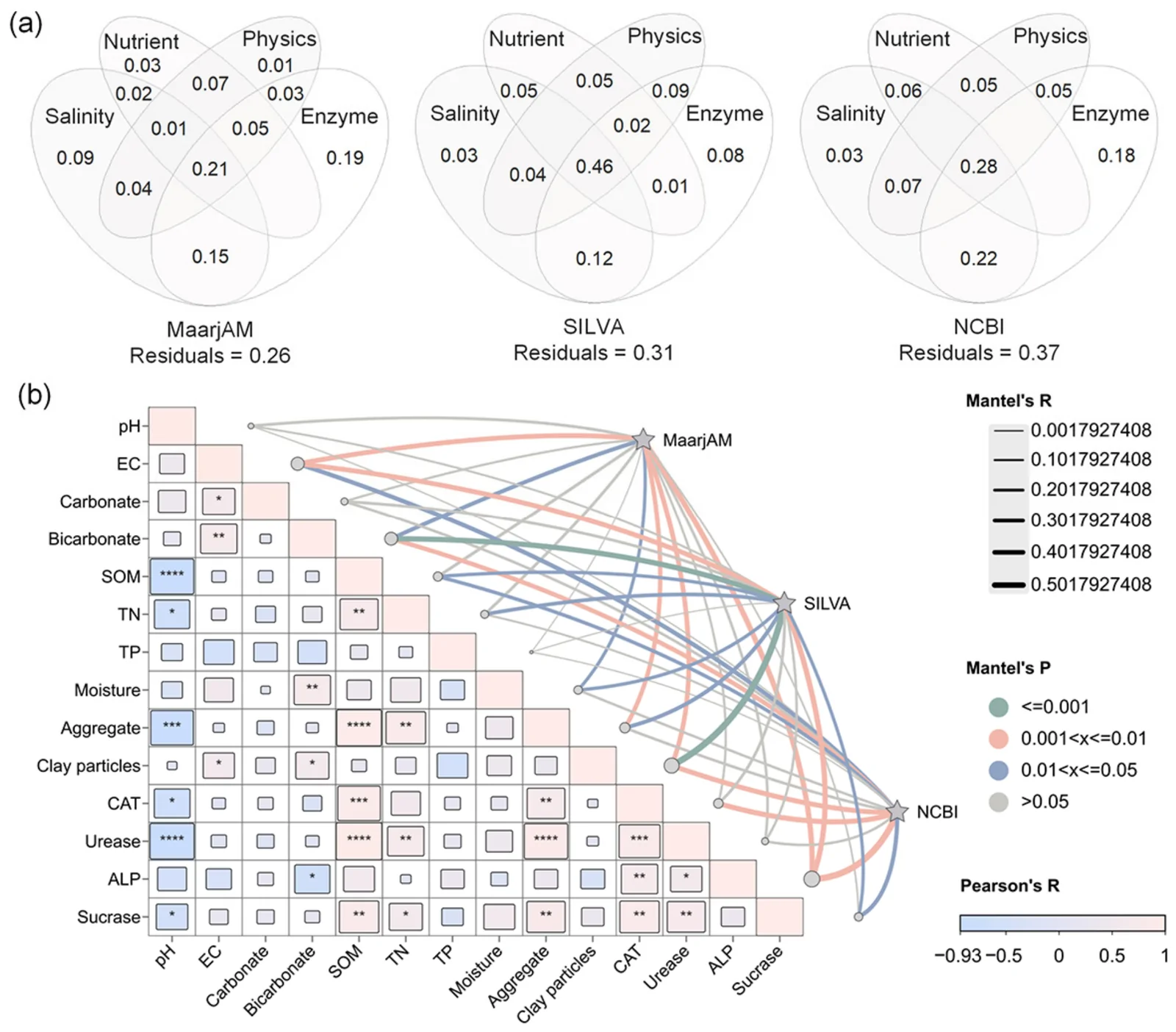
Associations between soil variables and AM fungal communities annotated using three reference databases: (**a**) VPA of soil factors and AM fungal communities. Numbers indicate the proportion of community variation explained by individual factors or their combined effects. (**b**) Mantel tests between soil factors and AM fungal community, *p* < 0.05 (*), *p* < 0.01 (**), *p* < 0.001 (***), and *p* < 0.0001 (****).

Identifying the factors driving community composition is an important aspect of ecological analysis. Given the differences among database-derived annotations, we further evaluated the influence of database selection on the relationship interpretation between community composition and environmental variables (Figure 5). Dimensionality reduction of soil environmental variables showed that the cumulative explained variance reached 83.7%, 92.42%, 83.01%, and 92.94% for soil salinization, nutrient accumulation, physical properties, and enzyme activities, respectively, indicating that the reduced variables adequately represented the overall variation within each environmental category and were suitable for subsequent analyses. VPA revealed that the four categories of environmental factors collectively explained 74%, 69%, and 63% of AM fungal community variation under MaarjAM, SILVA, and NCBI annotations, respectively (Figure 5a). Although the proportion of explained variation differed among database-derived datasets, all four environmental categories accounted for substantial portions of community variation across databases. Among the explanatory components, the combined effects of all environmental variables contributed the largest proportion of variation in all databases, followed by the interactive effects between soil salinity and enzyme activities. Taken together, all databases revealed significant associations between environmental factors and AM fungal community.

Subsequently, Mantel tests revealed distinct patterns in the associations between AM fungal communities and soil factors across the three databases (Figure 5b). While pH, CO_3_^2−^ concentration, and TP showed no significant correlations with any of the database-derived communities (*p* > 0.05), EC, HCO_3_^−^ concentration, clay content, and ALP were consistently associated with all three datasets (*p* < 0.05). The consistent relationships observed across databases suggest that these environmental factors may represent relatively stable drivers of AM fungal community composition. In contrast, associations involving the remaining environmental variables exhibited clear database-dependent patterns. MaarjAM-derived communities showed no significant associations with any soil nutrient parameters, whereas NCBI were significantly associated only with SOM, and SILVA showed significant relationships with both SOM and TN. Notably, catalase activity was exclusively correlated with the NCBI dateset, while SILVA uniquely correlated with all soil physical indicators. Collectively, although different databases can capture certain consistent environmental signals, their ability to identify specific environmental associations varies substantially.

## 4. Discussion

In this study, the physicochemical properties and enzyme activities of rhizosphere soils differed significantly among the four plant species. Specifically, likely due to the nitrogen fixation capacity of legumes and their ability to improve soil aggregation through root exudates and hyphal networks [35,36], enhanced nutrient accumulation and improved soil structure were observed in the rhizosphere of *M. sativa*. In contrast, consistent with the relatively rapid organic matter turnover commonly reported in wetland ecosystems [37,38], *L. salicaria* exhibited comparatively lower soil organic matter content. The rhizosphere soils of *I. japonica* were characterized by higher salinity and clay content, whereas *T. mongolicum* showed relatively high ALP activity. These edaphic differences indicate that the selected plant species represent distinct ecological niches, supporting the ecological representativeness of the sampling design and providing a solid basis for evaluating the effects of reference database selection on the interpretation of relationships between AM fungal communities and environmental factors.

Sequencing analyses revealed substantial differences among the three databases in AM fungal annotation and subsequent analysis, highlighting the influence of reference database selection on the robustness of study conclusions. Notably, despite some outdated taxonomic names, NCBI achieved the highest taxonomic resolution at both genus and species levels and successfully identified ecologically well-characterized species, including *R. intraradices* and *F. mosseae*. The roles of these species in enhancing plant nutrient acquisition and improving host stress tolerance have been widely documented [39,40,41], supporting the biological relevance of NCBI-based species. This advantage may be attributed to the comprehensive nature of the NCBI repository, where its extensive coverage increases the likelihood of matching sequences with diverse reference taxa [42]. Furthermore, morphological identification showed that, except for *G. pyriformis*, a unique lineage within Glomeromycota that does not establish symbiotic associations with plants [43], the remaining 30 ecologically relevant species annotated by the NCBI database were recovered through spore isolation. Meanwhile, the total number of morphologically identified taxa was comparable to that inferred from NCBI-based annotations. These support the consistency between molecular and morphological evidence and highlight the potential utility of NCBI-based annotations for ecological investigation. However, these morphological assessments should be interpreted with caution, as spore-based identification relies on expert observation and morphological judgment, which may introduce uncertainty and result in false-positive matches with sequencing-based annotations. Therefore, single-spore DNA sequencing should be incorporated in future studies as an important complementary approach for validating taxonomic assignments. Meanwhile, *A. schenckii*, which was annotated exclusively by the SILVA database, was not recovered through spore isolation. This discrepancy may reflect limitations in the annotation performance of the database; however, it could also arise from the inherent constraints of morphological identification, as spore sampling coverage may hinder the detection of low-abundance or rare species.

In contrast, although the MaarjAM database recovered the highest number of AM fungal sequences and ASVs, the proportion of taxa with ecological interpretability remained limited, consistent with previous observations by Stefani et al. [44]. These findings suggest that MaarjAM may have limited advantages for accurate taxonomic identification but remains sensitive for ASV-level analyses. In addition, a substantial proportion of taxa were assigned to *Glomus*, reflecting a well-documented taxonomic bias within this database. This pattern is largely attributable to the early classification framework underlying MaarjAM, in which most species producing “*Glomus*-like” spores were grouped into a broadly defined *Glomus* lineage. Consequently, taxa currently classified as *Funneliformis* or *Rhizophagus* were frequently misassigned in earlier systems [45]. Previous studies have reported that up to 85% of sequences may be assigned to the broad *Glomus* group when directly compared against MaarjAM [44], further quantitatively supporting the strong *Glomus* bias observed in this study and indicating that this limitation may be widespread across different ecosystems and primer systems. Importantly, such database-derived biases may not only obscure accurate interpretation of community composition but also affect identification of key taxa and introduce substantial errors in analyses conducted at genus/species levels. Addressing these limitations will require continuous updates and refinement of taxonomic information within reference databases. As another reference database, SILVA provides broad taxonomic coverage across diverse microbial lineages but showed limited annotation depth for AM fungi, resulting in the fewest species-level assignments in this study. This limitation is likely associated with its design as a general-purpose rRNA database rather than a database specifically optimized for AM fungal identification. Consequently, SILVA generally provides reliable annotations for microbial groups with abundant reference sequences and well-established taxonomies, while species-level resolution is limited for groups such as AM fungi with insufficient high-quality references and complex taxonomic histories [46]. Compared with comprehensive repositories such as NCBI, SILVA emphasizes sequence quality control and taxonomic consistency, which also restrict fine-scale assignments for poorly represented taxa.

The differential performance among databases was also reflected in alpha diversity indices, NMDS ordination, and Venn analyses. Consistent with its higher taxonomic resolution, NCBI database demonstrated greater sensitivity in capturing community richness, diversity, and evenness, as well as stronger sample separation in NMDS space, whereas the resolving power of MaarjAM and SILVA progressively decreased. This indicates that, under the same biological composition of the samples examined in this study, database-specific biases in species detection and taxonomic assignment substantially influenced the ecological interpretation of AM fungal community. Because niche breadth is an important metric for understanding community assembly, species coexistence, and ecosystem functioning, it was further evaluated across the database-derived datasets. The NCBI database showed a clear advantage, with species distributions across all samples closely matching ecological expectations: neutral specialists predominated, whereas specialists and generalists accounted for smaller proportions [47]. In contrast, both MaarjAM and SILVA showed marked deviations in several samples. These discrepancies suggest that inappropriate database selection may distort estimated niche breadths and lead to biased assessments of community specialization or generalization. This indicates that NCBI may better capture ecological patterns of AM fungal communities, whereas other databases could obscure some meaningful results. For functional pathway prediction, although differences in pathway abundances were observed among the three databases as a consequence of distinct input datasets, the core metabolic framework remained largely conserved. This supports previous evidence of substantial conservation in microbial basal metabolic functions across taxonomic assemblages [48], suggesting that database-dependent differences in taxonomic annotation have limited effects on the interpretation of core functional profiles. This conservation may also indicate functional redundancy within AM fungal communities, potentially contributing to more stable outcomes in agricultural and ecological restoration applications than single inoculation. However, the interpretation of the functional pathway should be considered cautiously, as functional predictions based on short amplicon regions and PICRUSt2 may contain uncertainties, particularly given that PICRUSt2 was originally developed for bacterial 16S rRNA data [49]. Future studies should therefore validate these predictions using metagenomic or metatranscriptomic approaches.

Previous studies on AM fungal communities based on high-throughput sequencing have identified soil heterogeneity as a major driver of community differentiation [50,51,52]. In this study, VPA revealed similar community–environment relationships across databases, indicating that edaphic factors explained a substantial proportion of AM fungal community variation regardless of the reference database used. However, Mantel tests revealed discrepancies in the associations between AM fungal communities and individual soil variables across the three datasets. This suggests that inappropriate database selection may substantially influence the identification of specific ecological drivers and thereby affect the interpretation of AM fungal community assembly processes. Among all environmental factors, EC, HCO_3_^−^ concentration, clay content, and ALP were consistently and significantly associated with AM fungal communities across all databases, indicating their broad importance in shaping community structure, consistent with previous studies [53,54,55,56]. Conversely, the relationships between AM fungal communities and other environmental variables varied among databases in both the number and strength of significant associations. Because all datasets were derived from the same sequencing results, these differences likely reflect variation in reference sequence coverage, taxonomic resolution, and species detection capability among databases rather than true changes in community composition. Such discrepancies may lead to biased interpretation or loss of important ecological signals [57,58], affecting downstream analyses including community structure, diversity patterns, niche differentiation, and identification of environmental drivers [57,59]. Specially, SILVA showed stronger associations with soil nutrients and physical properties and NCBI was more closely related to enzyme activities, whereas MaarjAM exhibited fewer significant environmental relationships overall. Given the superior annotation performance of NCBI, we speculate that its environmental associations may provide more reliable ecological insights and therefore recommend its preferential use for community–environment relationship analyses. However, the ecological relevance of individual environmental factors varies among studies [60,61], and current evidence remains insufficient to provide definitive explanations. Future research should therefore incorporate single-factor experiments to further validate the reliability of database-dependent ecological associations.

Collectively, NCBI provides advantages for species-level analyses of AM fungal high-throughput sequencing data, whereas MaarjAM may offer broader sequence coverage. These differences highlight the importance of database selection for AM fungal resource surveys, biodiversity assessment, and applications in agricultural production and ecological restoration, where reliable annotation can facilitate mycorrhizal resource screening and reduce biases in identifying key taxa. However, given the limitations of current individual databases, further efforts should focus on integrating their strengths to develop AM fungal-specific database by combining biological information from type materials with corresponding sequence data, followed by continuous taxonomic refinement. Notably, although this study employed morphological approaches as complementary validation methods, their inherent limitations may introduce uncertainty into the interpretation of actual community structure and potentially provide inaccurate reference for sequence-based analyses. In addition, this study was limited by the use of samples from a single region and a single amplicon marker, which may restrict the generalizability of our findings. Therefore, future studies should first evaluate database performance using mock communities with known species compositions and abundances, followed by validation with larger datasets spanning diverse ecosystems and multiple molecular markers to further improve the robustness and applicability of conclusions.

## 5. Conclusions

This study compared the performance of MaarjAM, SILVA, and NCBI databases for species-level analysis of AM fungal communities. NCBI provided higher species resolution and showed advantages in diversity estimation, sample discrimination, species screening, and niche analysis. These findings demonstrate that database selection influences not only taxonomic annotation but also downstream ecological analyses, with potential implications for biodiversity assessment, identification of key taxa, and ecosystem function studies. However, NCBI annotations should be validated using MycoBank. For studies focusing on community alpha diversity, MaarjAM remains a valuable resource, although its advantages may be more pronounced in AM fungal sequence detection and ASV-level characterization. In contrast, SILVA has limited applications due to its lower species-level resolution; nevertheless, its derived community datasets can still capture environmental associations and maintain relatively stable core functional predictions. Future efforts should integrate the strengths of multiple databases to develop reference resources with both broad AM fungal sequence coverage and high taxonomic accuracy, thereby improving species identification and ecological interpretation of AM fungal communities.

## Figures and Tables

**Table 1:** Rhizosphere soil properties of the four studied plant species.

	Indices	*M. sativa*	*I. japonica*	*L. salicaria*	*T. mongolicum*
Salinity	pH	7.54 ± 0.16 b	8.06 ± 0.19 a	7.63 ± 0.16 b	7.58 ± 0.19 b
	EC mS·cm^−1^	0.13 ± 0.02 b	0.19 ± 0.02 a	0.14 ± 0.02 b	0.13 ± 0.02 b
	CO_3_^2−^ cmol·kg^−1^	0.12 ± 0.02 b	0.25 ± 0.04 a	0.12 ± 0.02 b	0.23 ± 0.07 a
	HCO_3_^−^ cmol·kg^−1^	0.41 ± 0.05 b	0.58 ± 0.09 a	0.46 ± 0.04 b	0.33 ± 0.11 c
Nutrient	SOM g·kg^−1^	78.65 ± 1.85 a	38.29 ± 3.66 b	11.77 ± 4.56 d	28.20 ± 5.39 c
	TN g·kg^−1^	2.03 ± 0.51 a	1.42 ± 0.26 b	0.96 ± 0.10 c	0.44 ± 0.09 d
	TP g·kg^−1^	0.25 ± 0.07 a	0.18 ± 0.02 b	0.24 ± 0.03 a	0.24 ± 0.03 a
Physical property	Moisture %	8.66 ± 1.40 ab	9.22 ± 2.63 a	7.33 ± 0.98 bc	6.32 ± 0.27 c
	Stable aggregate %	52.90 ± 5.30 a	32.97 ± 4.74 b	17.64 ± 4.83 c	18.31 ± 3.81 c
	Clay particles %	24.83 ± 8.05 b	35.78 ± 7.73 a	23.33 ± 4.92 b	18.00 ± 6.18 b
Enzyme	CAT mL·g^−1^·20 min^−1^	2.55 ± 0.14 a	1.90 ± 0.19 b	1.15 ± 0.24 c	2.01 ± 0.10 b
	Urease mg·g^−1^·d^−1^	1.70 ± 0.24 a	0.98 ± 0.19 b	0.68 ± 0.15 c	0.88 ± 0.17 b
	ALP mg·g^−1^·d^−1^	2.58 ± 0.67 a	0.91 ± 0.35 b	0.45 ± 0.34 b	2.74 ± 0.59 a
	Sucrase mg·g^−1^·d^−1^	0.15 ± 0.04 a	0.14 ± 0.02 a	0.05 ± 0.01 c	0.09 ± 0.01 b

Values are presented as mean ± standard deviation (SD) of three replicates. Different letters within the same row indicate significant differences among groups (*p* < 0.05).

**Table 2:** Corrected taxonomic information for genera and species.

	MaarjAM	SILVA	NCBI
*Glomus*	VTX	VTX	*G. macrocarpum*
*Paraglomus*	VTX	/	*P. occultum*
			*P. majewskii*
*Ambispora*	VTX	*A. fennica*	*A. fennica*
			*A. reticulata*
			*A. leptoticha*
*Diversispora*	VTX	*D. eburnea*	*D. trimurales*
			*D. aurantia*
			*D. celata*
			*D. spurca*
			*D. nevadensis*
*Archaeospora*	VTX	*A. schenckii*	VTX
*Acaulospora*	VTX	VTX	VTX
*Rhizophagus*	/	VTX	*R. intraradices*
			*R. irregularis*
*Septoglomus*	/	VTX	*S. constrictum*
			*S. africanum*
			*S. furcatum*
*Funneliformis*	/	*F. mosseae*	*F. mosseae*
			*F. geosporum*
*Entrophospora*	/	*E. lamellosa*	*E. lamellosa*
			*E. etunicata*
		*E. etunicata*	*E. lutea*
			*E. claroidea*
*Scutellospora*	/	/	VTX
*Microkamienskia*	*M. perpusilla*	/	*M. perpusilla*
*Dominikia*	*D. iranica*	/	*D. gansuensis*
			*D. indica*
			*D. iranica*
*Sclerocystis*	/	/	VTX
*Geosiphon*	/	/	*G. pyriformis*
*Gigaspora*	/	/	*G. margarita*
			*G. decipiens*
*Blaszkowskia*	/	/	*B. deserticola*
*Viscospora*	/	/	*V. viscosa*

/ indicates that no sequences were assigned to the corresponding genus. VTX indicates that the genus was represented only by virtual species. Genera annotated with a species name also contained a large number of virtual species assignments.

## Data Availability

The raw sequencing data have been deposited in the NCBI Sequence Read Archive (SRA) under BioProject accession number PRJNA1495210.

## Appendix A

**Table A1:** Annotation results of sequencing data based on different databases.

	MaarjAM	SILVA	NCBI
*Glomus*	*G. iranicum*	VTX	*G. indicum*
	*G. perpusillum*		*G. macrocarpum*
*Paraglomus*	VTX	/	*P. occultum*
*Claroideoglomus*	VTX	*C. lamellosum*	/
		*C. etunicatum*	
*Ambispora*	VTX	*A. fennica*	*A. reticulata*
			*A. fennica*
			*A. leptoticha*
*Diversispora*	VTX	*D. eburnea*	*D. trimurales*
			*D. aurantia*
			*D. celata*
			*D. spurca*
*Archaeospora*	VTX	*A. schenckii*	VTX
*Acaulospora*	VTX	VTX	VTX
*Rhizophagus*	/	VTX	*R. iranicus*
			*R. irregularis*
			*R. intraradices*
*Septoglomus*	/	VTX	*S. deserticola*
			*S. viscosum*
			*S. constrictum*
			*S. furcatum*
			*S. africanum*
*Funneliformis*	/	*F. mosseae*	*F. mosseae*
			*F. geosporum*
*Entrophospora*	/	/	*E. lamellosa*
			*E. etunicata*
			*E. claroidea*
			*E. lutea*
*Scutellospora*	/	/	VTX
*Microkamienskia*	/	/	*M. perpusilla*
*Dominikia*	/	/	*D. gansuensis*
*Tricispora*	/	/	*T. nevadensis*
*Sclerocystis*	/	/	VTX
*Innospora*	/	/	*I. majewskii*
*Geosiphon*	/	/	*G. pyriformis*
*Gigaspora*	/	/	*G. margarita*
			*G. decipiens*

/ indicates that no sequences were assigned to the corresponding genus. VTX indicates that the genus was represented only by virtual species. Genera annotated with a species name also contained a large number of virtual species assignments.

**Table A2:** Taxonomic corrections based on the latest classification information.

Latest Update	MaarjAM	SILVA	NCBI
*Dominikia iranica*	*Glomus iranicum*	—	*Rhizophagus iranicus*
*Dominikia indica*	—	—	*Glomus indicum*
*Microkamienskia perpusilla*	*Glomus perpusillum*	—	√
*Entrophospora*	*Claroideoglomus*	*Claroideoglomus*	√
*Entrophospora lamellosa*	—	*Claroideoglomus lamellosum*	√
*Entrophospora etunicatum*	—	*Claroideoglomus etunicatum*	√
*Blaszkowskia deserticola*	—	—	*Septoglomus deserticola*
*Viscospora viscosa*	—	—	*Septoglomus viscosum*
*Diversispora nevadensis*	—	—	*Tricispora nevadensis*
*Paraglomus majewskii*	—	—	*Innospora majewskii*

— indicates that the species was not identified in the annotation results of the corresponding database; √ indicates that the species name assigned by the database is consistent with its currently accepted nomenclature.

**Table A3:** Morphological criteria for the identification of isolated AM fungal spores.

	Diameter	Color	Other
*G. macrocarpum*	110–120	yellow	Spores formed in clusters
*P. occultum*	60–100	hyaline to white	Outer surface often covered with debris; orange-yellow in Melzer’s reagent
*P. majewskii*	35–78	hyaline	Surface smooth to slightly roughened; subtending hypha narrow at base
*A. fennica*	150–200	hyaline to yellow	Spore wall often separating; red in Melzer’s reagent
*A. reticulata*	87–150	yellow brown	Spore wall with reticulate ornamentation and polygonal depressions
*A. leptoticha*	70–120	hyaline to pale yellow	Subtending hypha cylindrical to funnel-shaped, slightly expanded at base
*D. trimurales*	70–120	pale yellow	Subtending hypha hyaline; spore wall folded after rupture
*D. aurantia*	70–120	yellowish white	Spore wall transparent with mucilaginous granules; subtending hypha straight or recurved
*D. celata*	53–160	creamy white	Surface frequently covered with debris; subtending hypha long; pore closed
*D. spurca*	60–120	subhyaline	Dull-colored spores with debris; thick L2 wall layer
*D. nevadensis*	80–150	yellow to yellow brown	Sporocarps present; spore surface with verrucous projections
*D. eburnea*	40–120	pale cream	Larger spores often transparent; spore wall thin and prone to folding
*R. intraradices*	40–140	pale cream to yellow brown	Spore wall multilayered, dark red in Melzer’s reagent; subtending hyphal pore flame- to funnel-shaped
*R. irregularis*	70–165	hyaline to yellow brown	Spores subglobose to irregular; subtending hypha constricted at attachment
*S. constrictum*	130–180	red-brown	Subtending hypha dichotomously branched; constricted and thick-walled at attachment point
*S. africanum*	80–150	pale yellow	Spore wall smooth; subtending hypha slightly expanded at base
*S. furcatum*	120–165	reddish brown	Subtending hyphal vertically branched; pore septate
*F. mosseae*	100–260	yellow-brown	Pink in Melzer’s reagent; subtending hypha funnel-shaped at attachment
*F. geosporum*	120–240	yellow-brown	Light red in Melzer’s reagent; subtending hypha hyaline, thick and rigid, gradually tapering downward
*E. lamellosa*	80–140	pale yellow	Spore wall separating upon rupture; pale pinkish white in Melzer’s reagent
*E. etunicata*	75–135	pale yellow to yellow	Subtending hyphal pore blocked; opening cylindrical to funnel-shaped
*E. lutea*	60–180	pale yellow	Spore surface smooth; subtending hypha distinctly expanded at base, funnel-shaped
*E. claroidea*	80–160	pale yellow	Spore wall separation evident after rupture; pink in Melzer’s reagent
*M. perpusilla*	18–63	hyaline	Spore wall with inner and outer layers; reddish white to grayish red in Melzer’s reagent
*D. gansuensis*	20–86	pale yellow to yellow-brown	Spores often formed in clusters; spore wall smooth; subtending hypha slightly translucent
*D. indica*	30–70	hyaline to white	Spores often formed in clusters; spore wall smooth; pink in Melzer’s reagent
*D. iranica*	70–120	yellow	Spore wall distinctly layered, separating after rupture and forming folds; hypha constricted at attachment
*G. margarita*	260–400	white to cream	Dark reddish-brown in Melzer’s reagent; distinct globose sporogenous cells present
*G. decipiens*	300–420	yellow-brown	Bulbous cells present, paler and smaller than spores
*B. deserticola*	60–140	orange-brown	Surface slightly roughened; spore wall pale red and thickened at subtending hyphal attachment
*V. viscosa*	50–120	subhyaline	Spore wall separating after rupture; subtending hypha cylindrical

Values in the Diameter column are expressed in μm.

**Table A4:** Repeated-measures ANOVA for alpha diversity indices of AM fungal communities across different databases.

Index	Database	MarrjAM	SILVA	NCBI
Chao 1 partial η^2^ = 0.85 *	MaarjAM	—	2.23 *	0.18
	SILVA	−2.23 *	—	−2.05 *
	NCBI	−0.18	2.05 *	—
Simpson partial η^2^ = 0.43 *	MaarjAM	—	1.73 *	−0.58
	SILVA	−1.73 *	—	−2.3 *
	NCBI	0.58	2.3 *	—
Pielou_e partial η^2^ = 0.33 *	MaarjAM	—	0.10	−1.18 *
	SILVA	−0.10	—	−1.28 *
	NCBI	1.18 *	1.28 *	—
Shannon partial η^2^ = 0.69 *	MaarjAM	—	1.51 *	−0.95 *
	SILVA	−1.51 *	—	−2.47 *
	NCBI	0.95 *	2.47 *	—

Values in the table represent Cohen’s d values; * indicates significant differences between the corresponding groups.
